# Adsorption Behavior of Methylene Blue Cationic Dye in Aqueous Solution Using Polypyrrole-Polyethylenimine Nano-Adsorbent

**DOI:** 10.3390/polym14163362

**Published:** 2022-08-17

**Authors:** Abdullahi Haruna Birniwa, Habibun Nabi Muhammad Ekramul Mahmud, Shehu Sa’ad Abdullahi, Shehu Habibu, Ahmad Hussaini Jagaba, Mohamad Nasir Mohamad Ibrahim, Akil Ahmad, Mohammed B. Alshammari, Tabassum Parveen, Khalid Umar

**Affiliations:** 1 Department of Chemistry, Sule Lamido University, Kafin-Hausa P.M.B 048, Nigeria; 2 Department of Chemistry, Faculty of Science, University of Malaya, Kuala Lumpur 50603, Malaysia; 3 Department of Polymer Technology, Hussaini Adamu Federal Polytechnic Kazaure, Kazaure P.M.B 5004, Nigeria; 4 Department of Chemistry, Faculty of Science, Federal University Dutse, Dutse P.M.B 7156, Nigeria; 5 Department of Civil and Environmental Engineering, Universiti Teknologi Petronas, Seri Iskandar 32610, Malaysia; 6 Materials Technology Research Group (MaTRec), School of Chemical Sciences, Universiti Sains Malaysia, Gelugor 11800, Malaysia; 7 Chemistry Department, College of Sciences and Humanities, Prince Sattam bin Abdulaziz University, Al-Kharj 11942, Saudi Arabia; 8 Department of Botany, Aligarh Muslim University, Aligarh 202002, India

**Keywords:** adsorption, methylene blue, polyethyleneimine, polypyrrole, wastewater

## Abstract

In this work, a polypyrrole-polyethyleneimine (PPy-PEI) nano-adsorbent was successfully synthesized for the removal of methylene blue (MB) from an aqueous solution. Synthetic dyes are among the most prevalent environmental contaminants. A new conducting polymer-based adsorbent called (PPy-PEI) was successfully produced using ammonium persulfate as an oxidant. The PEI hyper-branched polymer with terminal amino groups was added to the PPy adsorbent to provide more effective chelating sites for dyes. An efficient dye removal from an aqueous solution was demonstrated using a batch equilibrium technique that included a polyethyleneimine nano-adsorbent (PPy-PEI). The best adsorption parameters were measured at a 0.35 g dosage of adsorbent at a pH of 6.2 and a contact period of 40 min at room temperature. The produced PPy-PEI nano-adsorbent has an average particle size of 25–60 nm and a BET surface area of 17 m^2^/g. The results revealed that PPy-PEI nano-composite was synthesized, and adsorption was accomplished in the minimum amount of time. The maximum monolayer power, qmax, for MB was calculated using the isothermal adsorption data, which matched the Langmuir isotherm model, and the kinetic adsorption data, which more closely fitted the Langmuir pseudo-second-order kinetic model. The Langmuir model was used to calculate the maximum monolayer capacity, or qmax, for MB, which was found to be 183.3 mg g^−1^. The as-prepared PPy-PEI nano-adsorbent totally removes the cationic dyes from the aqueous solution.

## 1. Introduction

The demand for water, food, agricultural products, and other natural resources has expanded dramatically as a result of industrial development, urbanization, and population growth. Various concerns about delivering clean and useable water have been raised across the world due to climate change, misuse and depletion of water supplies, and human-caused water pollution. Water contamination has become a public concern, posing a threat to ecosystems and human life security. Water pollution from different dyes has become a serious concern to human and marine life in recent years [1,2]. There are various types of dyes used in the pulp, textile, clothing, fabrics, food, and pharmaceutical cosmetics sectors can pose major problems by impeding sunlight penetration into the water and harming aquatic life [3,4,5]. Complex aromatic molecules are more stable and difficult to biodegrade than many artificial dyes [5,6]. Some dyes have been linked to allergies, skin irritation, and even cancer in humans [7,8,9]. Therefore, environmental scientists and engineers must still determine how to remove dyes effectively and comprehensively from water sources [10].

The most prevalent dye is methylene blue (MB), which is used to dye wool, silk, and cotton, among other things. methylene blue adverse effects include eye burning, methemoglobinemia, trouble breathing, hyperplasia, convulsions, tachycardia, skin and gastrointestinal system irritation, nausea, diarrhea, and vomiting [2,10,11]. Furthermore, several processes have been employed to remove dyes from wastewater or aqueous solutions, including reverse osmosis, membrane filtration, ion exchange, adsorption, electrochemical technologies, solvent extraction, chemical precipitation, and so on [12,13,14]. Except for the adsorption approach, none of these technologies are routinely employed since they are either expensive or ineffective. Adsorption has various advantages, including lower startup costs, easy design, and quick functioning operation [15]. Furthermore, at extremely low concentrations, the adsorption approach can eliminate particular or target contaminants [16].

A large variety of adsorbent materials such as carbon material [17], chitosan [18,19], nanocellulose [20], cellulose nanofibrils (CNFs), palm oil agriculture waste [21,22,23], cotton stalk [24], jackfruit leaves [25], guava leaves [26], yellow passion fruit waste [27], neem leaves powder [28], rice husk [29], and granular activated carbon (GAC) have been tested in the removal of different dyes [30]. Because of their refractory and dangerous nature, many dyes are not easily degradable and hence remain in water supplies for prolonged periods of time [31]. Adsorption will provide high-quality water while being a cost-effective solution. [32]. Nanomaterials and polymers are among the most promising new materials for wastewater treatment. They have distinct functional features that may be tailored to meet the needs of a variety of wastewater treatment systems. Polymer nanocomposites are a specialized group of substances that provide greater performance by combining the advantageous features of polymers with nanoparticles. Of course, the creation of these kinds of functional materials necessitates a thorough grasp of their fundamental features and the underlying science. Due to the presence of specific functional groups that might mix well with diverse contaminants, polymers, for example, polypyrrole (PPy) and polyaniline, as well as their composites, are getting broad consideration in ecological effluent remediation [33]. For the clearing of various harmful dyes, such as Congo red, malachite green, receptive blue, carmoisine, and others, some of these polymeric materials have already been examined for their activity, ease of combination, and the existence of a recovery connection [34]. Because of their ease of synthesis, redox characteristics, and biocompatibility, PPy-based adsorbents have sparked a lot of scientific interest [35]. The removal of dyes and their components by polypyrrole-directing polymer-based adsorbents has been accounted for inconsistently, and scarcely any investigations have reported it [34,36]. The use of composites made up of PPy and other materials for the elimination of reactive red dye from an aqueous solution was reported in one study using -cellulose/polypyrrole composites [34]. Recent studies using PPy/TiO_2_ MB dyes as a nano-adsorbent have shown good adsorption capacity [34]. The fibrous adsorbents and decontaminated water might be effortlessly isolated after the adsorption interaction since they have an enormous explicit surface region, making adsorption on these adsorbents speedy and productive. Fibrous adsorbents have been created as a reasonable option in contrast to run-of-the-mill adsorbents like enacted carbon in the last ten years. Fibrous adsorbents are preferred to conventional adsorbents because of their exceptionally active surface, satisfactory mechanical strength, surface chemical change, pore size dispersion, and reusing capability [37]. Fibrous filters’ resistance to the flow of wastewater may be readily regulated. Several investigations have been conducted on the modification and conversion of typical textile fibers into adsorbent structures capable of removing various substances from aqueous environments, including textile dyes, heavy metal ions, and other hazardous compounds. The dye removal efficiency of polypropylene (PP) nonwoven fabric was 88.72 percent [38], and acrylic fibers modified with NaOH and citric acid are able to remove some selected heavy metal ions from water efficiently [39]. The use of ethanolamine and diethylenetriamine to modify acrylic fibers led in the creation of chelating fibers capable of removing Cr^6+^, Pb^2+^, Cu^2+^, Ce^3+^, and Ni^2+^ from aqueous environments [40,41]. Polyester fibers prepared with formaldehyde and grafted with methacrylic acid demonstrated the capacity to adsorb Cu^2+^ and Pb^2+^ from water [42]. Polyvinylamine-modified polyester fibers successfully eliminated Cr^6+^ from polluted groundwater. Previously, an adsorbent was prepared by grafting glycidyl methacrylate onto PET fibers and functionalized with iminodiacetic acid, which demonstrated good removal effectiveness with two cationic dye solutions [38].

Although attempts have been made to recover MB from wastewater using PPy alone, which has a poor adsorption mechanism for dyes and heavy metals, no attempt has been made to extract methylene blue using PPy-PEI [43]. In the present study, a conducting polymer of polypyrrole (PPy) and hyper-branched polymer (with terminal amino groups) polyethyleneimine (PEI) formed (PPy-PEI) adsorbent was synthesized for the elimination of harmful cationic methylene blue from commercial effluent. The adsorbent’s preparation conditions were crucial in the creation of an effective adsorbent for methylene blue dyes from a watery solution. The effect of numerous limits on the adsorption’s working conditions, such as adsorbent dosage, contact duration, beginning pH, and MB dye concentration, was explored, as well as the adsorption of isotherms and kinetic properties of methylene blue dye adsorption on the PPy. The synthesized adsorbent is environmentally friendly, efficient, and cost-effective, and it can readily be scaled up to large-scale use without causing harm to the environment or human health. 

## 2. Experimental

### 2.1. Materials

**Figure 1:**
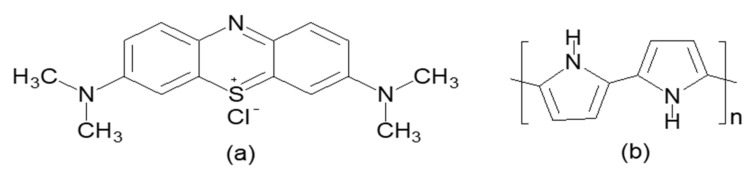

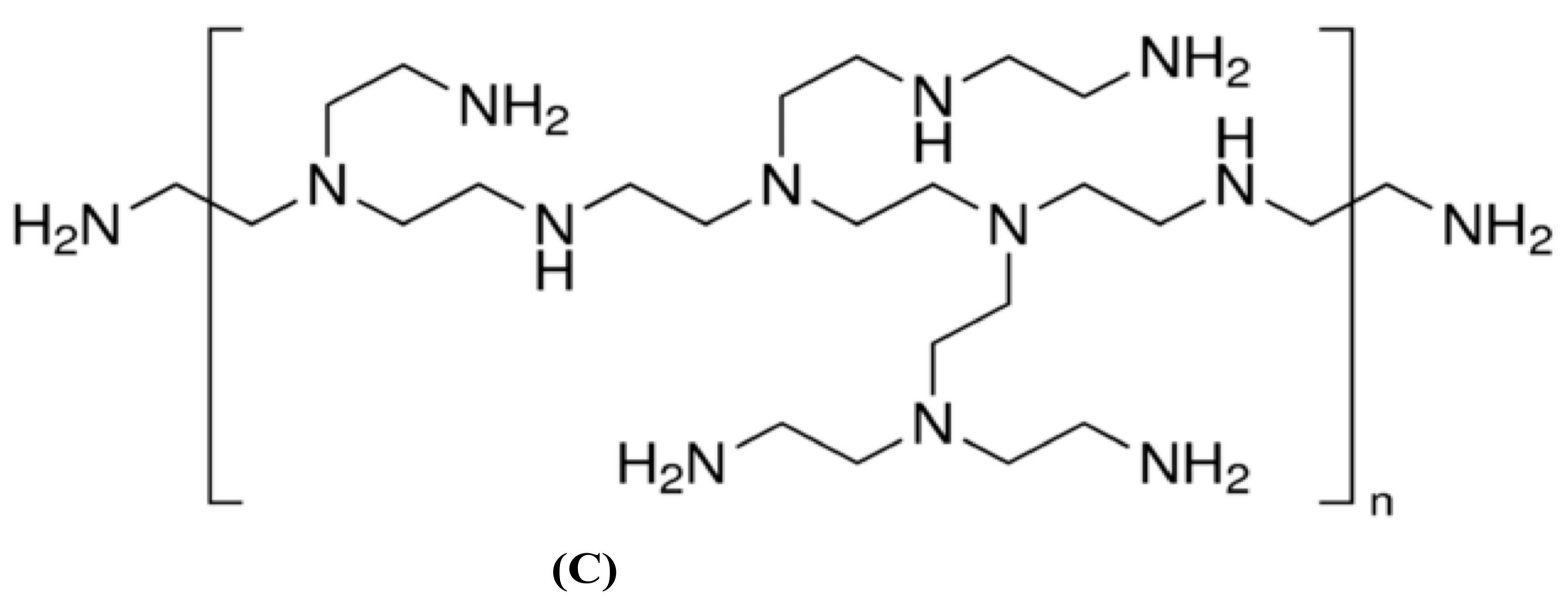
Molecular structure of (**a**) MB (an adsorbate) and (**b**) PPy (adsorbent) and (**c**) (PEI).

Methylene blue Sigma Aldrich (St. Louis, MO, USA), dye was used as the dyeing agent (adsorbate) in the formulation of a stock solution with the formula C_16_H_18_N_3_SCl, as illustrated in Figure 1. Diluting 1.0 g of MB powder in a 1000 mL volumetric flask filled with distilled deionized H_2_O yielded a 1000 mg/L MB stock solution. Dilutions of the stock solution were used to make the methylene blue -treated solutions with the appropriate concentration.

The monomer, pyrrole (99 percent), was distilled (purified) before use, and the oxidizing agent was ammonium persulfate (NH)_2_S_2_O_8_. Polyethyleneimine (molecular weight: 1200–1300) was combined with distilled PPy to generate PPy-PEI polymer nano-adsorbent. methylene blue stock solution was prepared with distilled-deionized H_2_O; many of the reagents were also provided by (Sigma-Aldrich) USA. Merck New Jersey USA provided the HCl and NaOH necessary to adjust the pH.

### 2.2. Polypyrrole and Polypyrrole-Polyethyleneimine Synthesis

**Figure 2:**
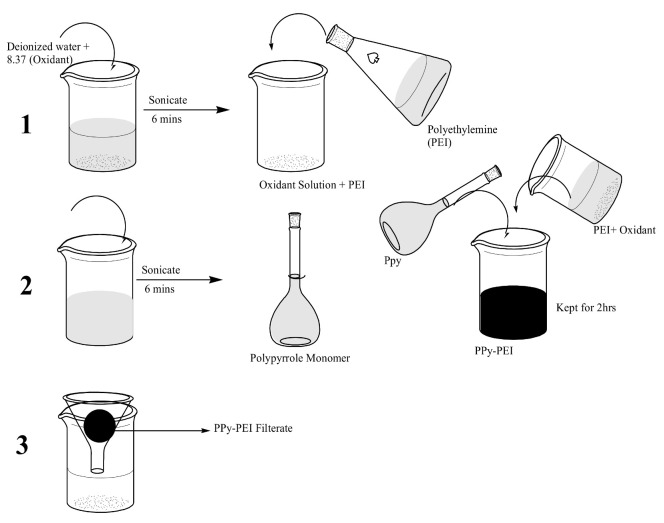
PPy-PEI nano-adsorbent’s synthesis design.

Based on the mole ratio of the oxidant to the monomer, the necessary amount of oxidant was thoroughly dissolved in deionized water using sonication for five minutes. Following that, the oxidant solution was combined with PPy solutions containing PEI and slowly shaken for three hours at room temperature. A shift in the dye of the solution from light green to black showed the growth of the products (PPy-PEI). The mixture’s black substance was filtered, washed several times with deionized water, and then dried for 24 h at 65 °C to remove extra reactants. As shown in Figure 2, the polymer product was ground into a fine powder using a mortar and pestle before being sieved using a No. 120 US Standard Sieve to create a fine powder.

### 2.3. Characterization of PPy Nano-Adsorbents

A Perkin Elmer Spectrum spectrometer 400 (New Jersey, NJ, USA) equipped with an FTIR microscope attachment, and a germanium crystal was used to obtain the ATR-FTIR spectra of the PPy-PEI powder nano-adsorbent at ambient temperature.

#### 2.3.1. Surface Analysis

Using N_2_ adsorption on a surface analyzer, a Brunauer–Emmett–Teller (BET) surface study of PPy-PEI adsorbents was performed at 77.40 K. (1990, Sorptomatic Thermo Finnigan, Mundelein, IL, USA). The pore structure of this adsorbent was determined using the t-method.

#### 2.3.2. Thermal Property Measurement 

A TGA analyzer (STA 6000 Perkin Elmer, New Jersey, NJ, USA) with a temperature scope of 25 °C to 800 °C was utilized to play out the TGA-DTA investigation. The investigation utilized a nitrogen (N_2_) heating environment with a stream pace of 50 mL/min and a temperature pace of 10 °C/min. For the adsorbents utilized in adsorption, a Differential Scanning Calorimetry (DSC) (TA DCS Q20 V24.10) was utilized under encompassing nitrogen (20 cm^3^/min) with a warming pace of 10 °C/min.

#### 2.3.3. Gel Permeation Chromatography 

A Waters 2414 was loaded using a Gel Permeation Chromatography (GPC) device. D-CCl4 was used as the eluent at 40 °C with a stream rate of 1 mL min^−1^, and a refractive record identifier in order to calculate the molecular weight of the polypyrrole-polyethyleneimine nano-adsorbent. Utilizing a defined adjustment bend and restricted molecular weight polystyrene as a control, the Breeze register software was used to determine molecular weight.

#### 2.3.4. X-ray Diffraction (XRD)

X-ray diffraction (XRD) studies were carried out using an X-ray diffractometer (Brand: PAN analytical, model: Empyrean, Malvern, UK). Cu K radiation (=) was used to detect XRD patterns in the 2-range of 5–90 2theta with a phase length of 1.25 s.

#### 2.3.5. Morphological Analysis

The morphology of the PPy-PEI product was assessed using an energy-dispersive X-ray spectroscopy (EDS or EDX) equipped field emission scanning electron microscope (FE-SEM Type SU 8220 brand, Hitachi, Japan).

### 2.4. Batch Adsorption

Each experiment was run three times, and the mean was used to ensure accuracy. Batch adsorption was done utilizing the created PPy-PEI adsorbents at known methylene blue concentrations. Between 0.05 and 0.5 g/mL of adsorbent were employed in 25 mL of *MB* at known concentrations. When the platform shaker reached an ideal pH of 6.2 at room temperature and a rotating velocity of 150 rpm in the pH range 2.2–12.2, the effect of pH on *MB* adsorption was discernible. The initial *C*_0_ (mg/L) and final *C_e_* (mg/L) concentrations of methylene blue in this solvent at 664 nm were calculated using a UV-visible spectrophotometer (Shimadzu, 2600, Kyoto, Japan) (1).

(1) $$\%\;Removal\;of\;MB=\left\{\frac{C_{0}-C_{e}}{C_{0}}\right\}\times 100$$

(2) $$Q_{e}=\left\{\left(C_{0}-C_{e}\right)V\right\}/m$$

where *m* is the arrangement’s capacity in liters and *V* is the mass of the adsorbent in grams. In 10-min augmentations, the influence of contact time (from 0 to an hour) on *MB* adsorption was focused while all other parameters were maintained constant. Different initial *MB* fixations (10–40 ppm) were applied at different temperatures (20, 40, and 60 °C) at a range of adsorbent focuses, pH, and contact lengths in order to study the effects of temperature on *MB* adsorption.

## 3. Results and Discussion

### 3.1. Physical Characteristics of PPy-PEI 

**Figure 3:**
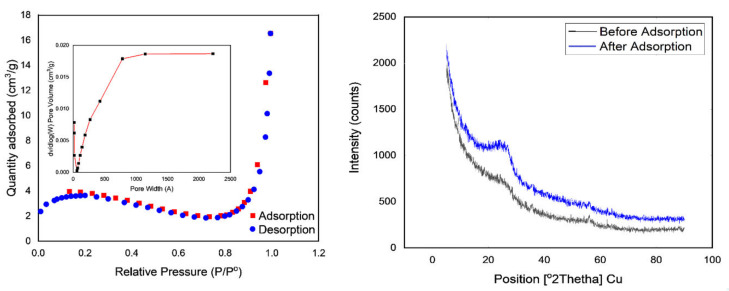
(**a**) N_2_ gas adsorption/desorption isotherms of PPy-PEI (**b**) X-ray diffraction of PPy-PEI before and after the adsorption process.

In PPy-PEI adsorption studies, the surface area is critical. Nitrogen adsorption–desorption experiments were performed to evaluate the porous nature and specific surface areas of the PPy-PEI nano-adsorbent material, as shown in Figure 3a. The primed nano-adsorbent PPy-PEI’s physical characteristics are as follows. Particle size was estimated to be between 25 and 60 nanometers. The generated PPy-PEI fine powder’s BET surface area, pore volume, and pore size were 17.04 m^2^/g, 0.017 cm^3^/g, and 79.9, respectively, as shown in Figure 3a. Following methylene blue adsorption, PPy-PEI exhibits a type-II sorption isotherm with saturation at a relative pressure (P/P0) of 0.3, the volume of holes reduced substantially to 0.018, the total quantity of N^2^ adsorbed at P/P0 1.0, and the BET surface area decreased to 13.94 m^2^/g from 17.04 m^2^/g. Furthermore, at a specific pressure of roughly 0.82, adsorption isotherms exhibit steep behavior, showing that mesopores and macropores exist on PPy/PEI [44]. The average adsorption pore size diameter ranged from 64 to 79 μm, demonstrating the microspore nature of the nano-adsorbent. The Ppy-PEI chelate was efficiently formed between the nano-adsorbent and the methylene blue, as evidenced by the decrease in surface area and pore volume [45].

Figure 3b shows the PPy-PEI adsorbent’s XRD diffraction type during methylene blue dye adsorption. The PPy-PEI displays a broad pinnacle focused at around 2θ = 26.12° prior to methylene blue adsorption, showing the hazy properties of the PPy-PEI nano-absorbent, which are primarily brought on by the equal periodicity of the polymer chain. The PPy-PEI adsorbent showed a virtually equivalent expanding top with enlarged force after methylene blue adsorption; however, it was seen to have shifted to a different location at 2θ = 25.04°. Even though the expanding pinnacles were not very transparent but did become more intense after the adsorption treatment, this adjustment of the expansive pinnacle combined with the PPy-PEI reveals MB adsorption on the adsorbent of PPy-PEI [46].

**Figure 4:**
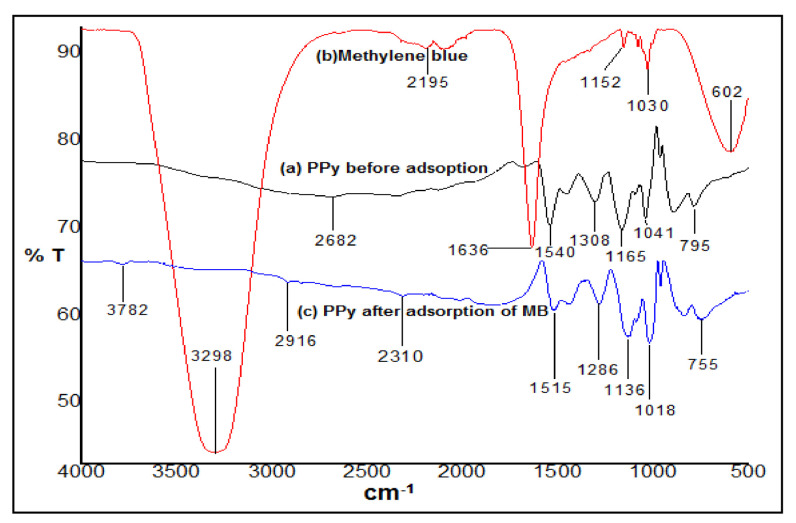
FTIR spectra of (**a**) PPy fine powder before adsorption, (**b**) *MB*, and (**c**) PPy after adsorption of methylene blue.

The ATR-FTIR spectra of (a) PPy fine powder prior to adsorption, (b) methylene blue, and (c) PPy following MB adsorption are shown in Figure 4. The usual PPy characteristic peaks before adsorption include the bands at 795 cm^−1^, 1041 cm^−1^, 1165 cm^−1^, 1308 cm^−1^, 1540 cm^−1^, and 2682 cm^−1^ [41]. A noticeable broad peak at 3298 cm^−1^ in 4b revealed the existence of OH and NH_2_ groups of methylene blue, whereas the acute peak at 1636 cm^−1^ was attributed to the unique aromatic rings. In the FTIR of methylene blue treated PPy (after adsorption) in Figure 4c, the enormous PPy peak at 2682 cm^−1^ (before adsorption) disappeared, and a few tiny peaks emerged at 2916 cm^−1^ and 2310 cm^−1^ in its place. Figure 4a shows that the bands at 1540 cm^−1^ attributable to C-N pyrrole ring straightening (before adsorption) shifted significantly to 1515 cm^−1^ after methylene blue adsorption [47] After methylene blue adsorption, the bands at 1308 cm^−1^ owing to C-N in-plane deformation and 1041 cm^−1^ due to N-H wagging migrated to 1286 cm^−1^ and 1018 cm^−1^, respectively.

**Figure 5:**
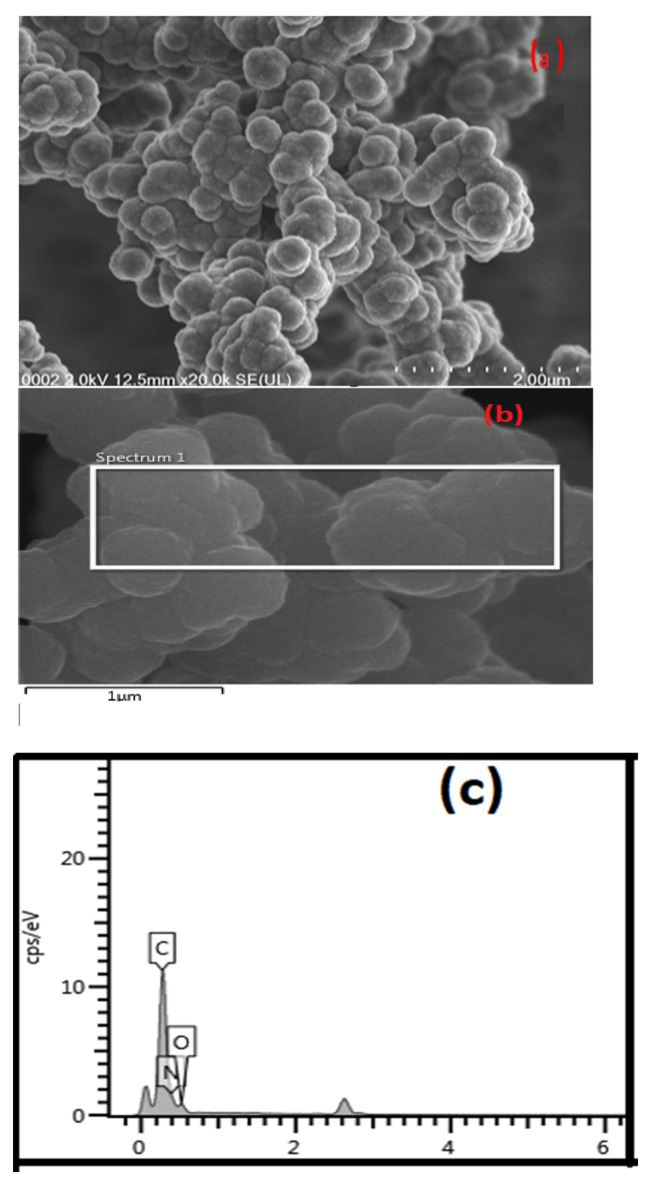
FESEM image of (**a**) PPy-PEI before and (**b**) EDX image of prepared PPy-PEI; (**c**) EDX graph showing elemental analysis of prepared Ppy-PEI.

Field emission scanning microscopy was used to analyze the morphology of polypyrrole-polyethylimine nano-adsorbent. The FESEM pictures revealed that PPy-PEI was successfully synthesized; it possesses tumor-like or cauli-like characteristics. Figure 5a depicts a micrograph of the polypyrrole-polyethyleneimine powder formed before adsorption with methylene blue, demonstrating that it was uniformly distributed over the polypyrrole surface, while Figure 5b shows the energy dispersion X-ray analysis of prepared PPy-PEI nano-adsorbent at 10 µm. Figure 5c, shows the graph of an EDX elemental analysis of the prepared nano-adsorbent. [48]. Because PPy-PEI has less aggregate formation, its adsorption to methylene blue is stronger, as is the fine powder’s small nano size [49]. Using the program “image J,” the average particle size was calculated by selecting a picture with a resolution of 2.0 µm. Figure 5 shows PPy-PEI average particle sizes ranging from 25 to 60 nm, with an average maximum particle size distribution of 35–40 nm [46,50].

### 3.2. Thermal Analysis

#### 3.2.1. Thermogravimetric Analysis (TGA)

**Figure 6:**
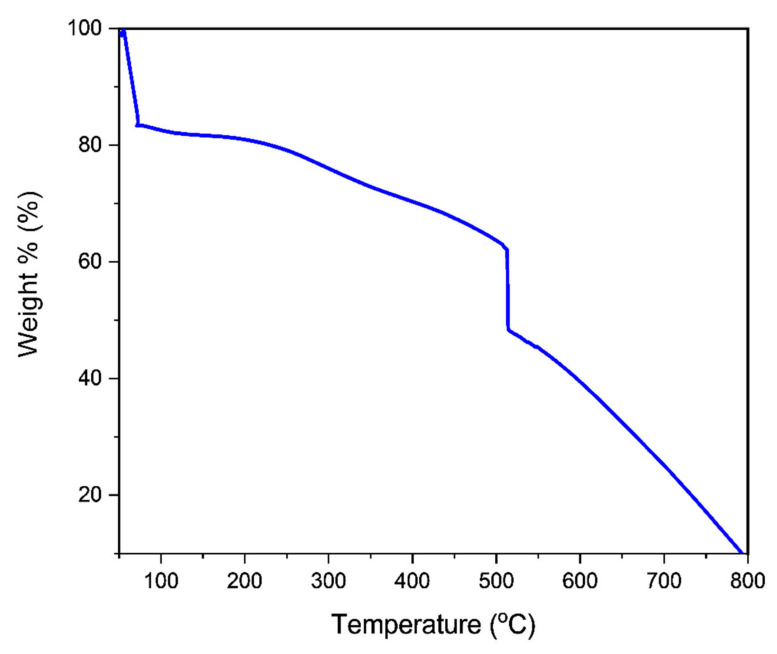
TGA graph representing the temperature-dependent thermal breakdown of PPy-PEI.

The temperature performance of the generated PPy-PEI adsorbent was investigated using TGA (PPy-PEI). Figure 6 shows the samples’ first weight loss, which begins at 50 °C and remains until nearly 200 °C, resulting in a 17.37 percent loss of polypyrrole-polyethyleneimine. The first lost mass at about 100 °C can be attributed to the sample’s moisture content (the boiling point of water). The second mass loss of the produced polypyrrole-polyethyleneimine was about 31% at 510 °C; this was related to both polypyrrole-polyethyleneimine nano-adsorbent breakdown and heat deterioration of the oligomeric or unsaturated group in the polymer nano-adsorbent. At 510 °C, the slope of the curve steadily reduced (50 percent weight loss). The heat breakdown of less than 46% of the initial polymer mass in the last step after 510 °C illustrates the strong thermal nature of the polypyrrole-polyethyleneimine nano-adsorbent [50].

#### 3.2.2. Differential Scanning Calorimetry (DSC)

**Figure 7:**
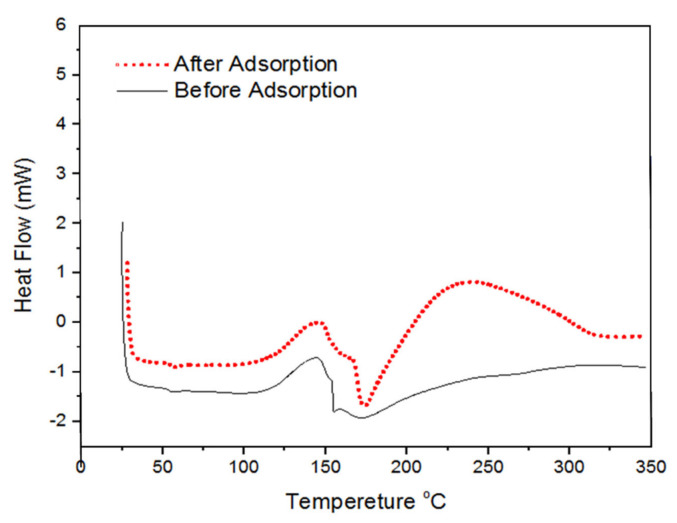
DSC curve of polypyrrole-polyethylimine.

A differential scanning calorimetry (DSC) study on the polypyrrole-polyethyleneimine adsorbent with and without adsorption treatment was carried out in a nitrogen environment at a heating rate of 10 °C min^−1^. The lack of a melting endothermic peak in the DSC curve of PPy-PEI nano-adsorbent in Figure 7 highlights the nano-adsorbent’s amorphous nature. The DSC thermogram for PPy-PEI showed a very wide exothermic peak at 240 °C after adsorption treatment, while no such wide-ranging peak was seen for polypyrrole-polyethyleneimine nano-adsorbent before adsorption treatment. This energy hump coupled with the PPy-PEI nano-adsorbent exhibits internal bond rearrangements in polymeric polymers containing methylene blue dyes after adsorption treatment, leading to energy release [46].

### 3.3. Gel Permeation Chromatography (GPC)

**Figure 8:**
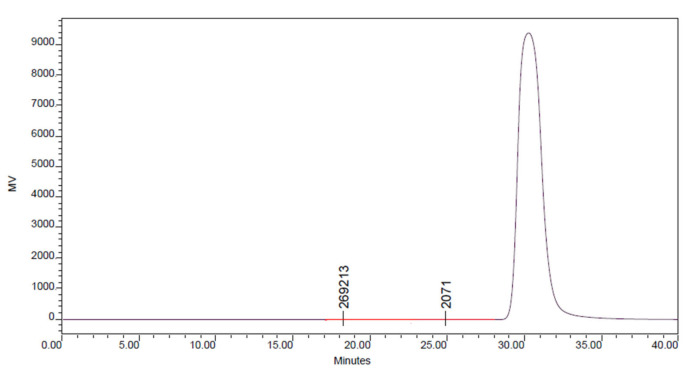
Distribution of Molecular Weight of PPy-PEI.

Gel permeation chromatography (GPC) was used to measure the molecular weight and molecular weight averages of the polymer as shown in Figure 8. (Water 2414 Refractive Index Detector) [51]. The total weight of the polymer was divided by the total mass number of molecules present, i.e., (Mn), and 1595 and 1578 were discovered for PPy-PEI, respectively. The average molecular weight (Mw) of PPy-PEI was 1934. The polydispersity index (PDI) for PPy-PEI was 1.21, suggesting that the polymer sample had a more evenly distributed dispersion of polymer chains. 

### 3.4. Effect of Adsorbent Doses

**Figure 9:**
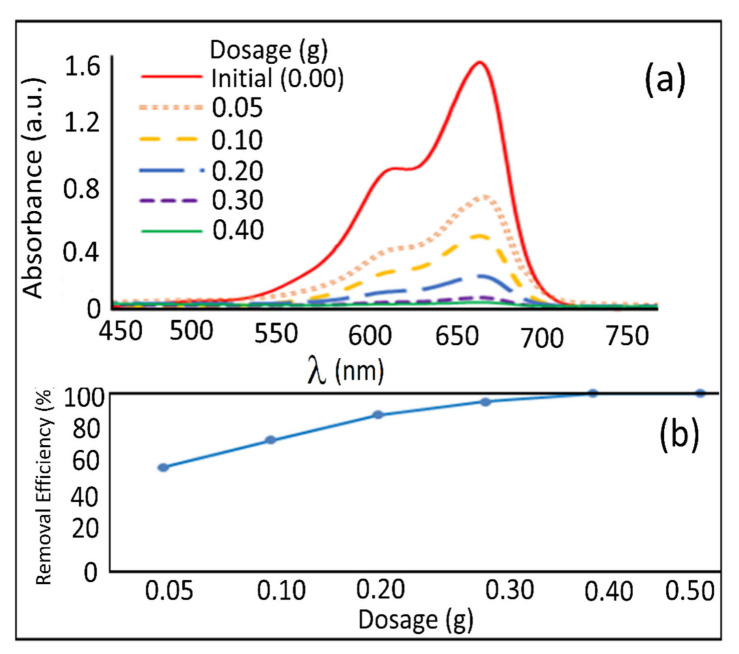
(**a**) UV-visible spectrometer and (**b**) methylene blue elimination effectiveness.

An UV-visible spectrophotometer was utilized to concentrate on the methylene blue dye adsorption by the PPy-PEI adsorbent at different portions. Figure 9a showcases the measurement reactions. It shows that as focus rises, the absorbance unit falls until it arrives at zero at the suggested level (0.35 g). As a result, as the adsorbent volume rises, the adsorption potential increases, corresponding to an increase in active sorption sites. Figure 9b illustrates the efficiency of the PPy-PEI adsorbent in removing MB from a 20-ppm starting methylene blue solution at 0.05 to 0.5 dosages. When 0.35 g of PPy-PEI was introduced, the MB was completely removed (100%) and no more adsorption occurred until the concentration reached 0.5 g, indicating saturation. Due to the existence of more active sites on the nano-adsorbent, the level of the end increased as the adsorbent part grew. The presence of extra hyper-branched end groups (NH_2_) on the PEI may have contributed to the end level rising when the adsorbent fraction was increased [52,53]. With increasing doses above saturation, however, there was no increase in the percentage of dye reduction. The amount of PPy-PEI in the body has no influence on methylene blue removal. The extra MB is most likely concealing some PPy-PEI adsorption sites, which explains why. Agglomeration when higher concentrations were used might be another explanation for the masking of some active sites of the PPy-PEI adsorbent. Others have observed that various adsorbents have similar adsorption tendencies for methylene blue dyes [12,36].

### 3.5. Impact of Initial pH

**Figure 10:**
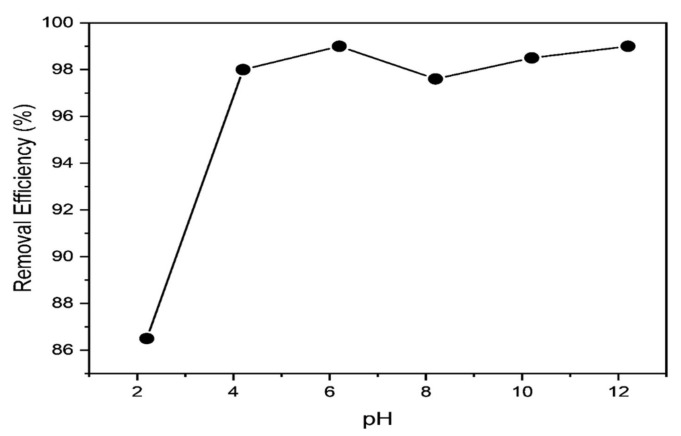
Impact of pH on MB adsorption.

One important factor that affects the adsorption process and provides important details regarding the adsorption mechanism is the initial pH of the adsorptive solution. Initial pH regulates (1) the dye’s charge by controlling the protonation/dissociation of the dye’s functional groups, (2) the adsorbent’s surface charge by managing the protonation/dissociation of the surface functional groups, and (3) the speciation degree of hydration of heavy metals. Because the adsorption process is initially pH dependent, electrostatic interactions and/or complexation processes must be present. Since it can impact the dye adsorption process by altering the surface charge of both the polypyrrole-polyethylimine and the methylene blue dye’s ionization workouts, the initial (underlying) pH plays a vital effect during the whole adsorption period. Figure 10 shows how beginning pH affects methylene blue adsorption on the PPy-PEI adsorbent. Because protons compete with dye particles for available adsorption sites, pH 2.2 caused PPy-PEI to have a reduced expulsion rate (86.4%). The amount of disposal increased significantly as the pH decreased, reaching its peak (100%) at pH 6.2. methylene blue elimination increased once again at a pH of 12.2, most likely because of MB cation precipitation. The methylene blue adsorption PPy-PEI adsorbent movement revealed a harmonic pH of 6.2 for optimal adsorption. methylene blue adsorption using natural palygorskite and polydopamine microspheres yielded similar results [54,55].

### 3.6. Impact of Contact Time

**Figure 11:**
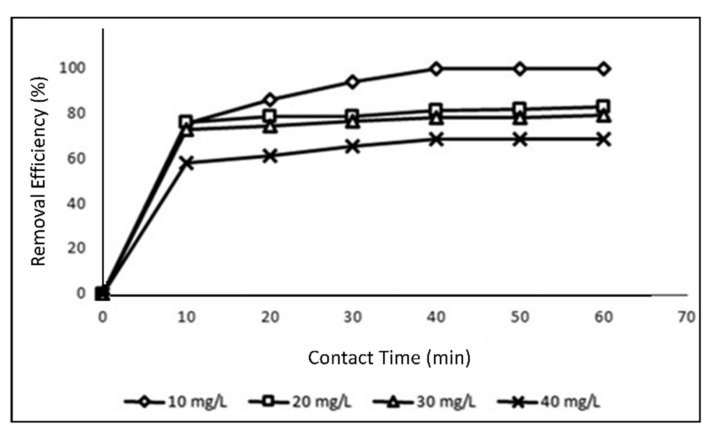
Effect of contact time on the effectiveness of methylene blue dye removal.

Using a 20 ppm methylene blue fixation at 20 °C in balance at pH 6.2, the effect of contact duration on adsorption term was evaluated (Figure 11). In the first 10 min, the adsorption rate of methylene blue was high showing that the molecules methylene blue and PPy-PEI had a strong affinity. Currently, over the surface, MB adsorption was almost homogeneous. After this point, the rate of adsorption gradually decreased until it reached equilibrium at 40 min, which may have been caused by long-term methylene blue dispersion into the nano-adsorbent PPy-PEI [56].

### 3.7. Impact of the Temperature

**Figure 12:**
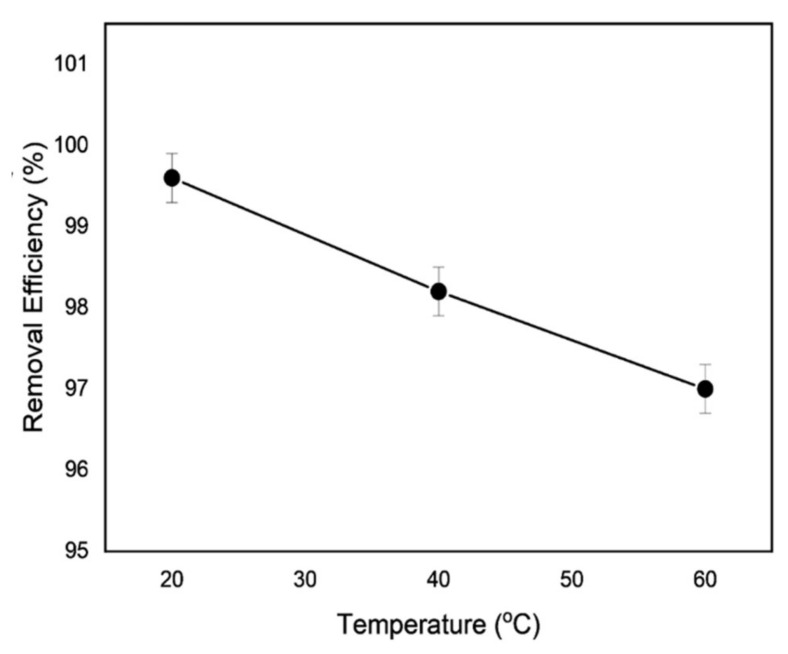
Shows how the adsorption of *MB* is impacted by temperature.

**Figure 13:**
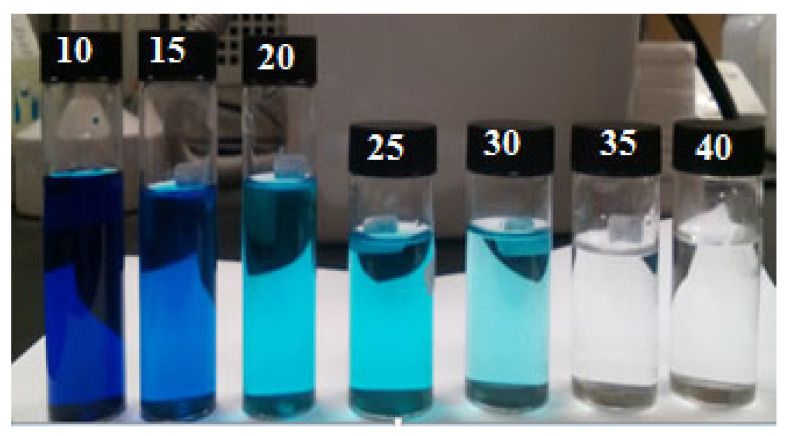
The images show *MB* solutions approaching adsorption equilibrium at 5-min intervals (10–40 min).

The effect of temperature on the adsorption of the adsorbent cycles was studied, as shown in Figure 12, by changing the arrangement temperature from 20 to 60 degrees Celsius. Adsorption productivity decreased with temperature, perhaps as a result of reduced surface mobility, proving that the methylene blue time of adsorption on the adsorbent polypyrrole-polyethyleneimine is exothermic. When employing other adsorbents, several studies found comparable methylene blue adsorption behavior. They linked the shorter sorption cycle to diminishing interactions between the methylene blue and the adsorbent in an advanced heat environment [57,58]. Figure 13 also illustrates how methylene blue adsorption occurred at 5-min intervals.

### 3.8. Adsorption Isotherm

The trial data was fitted using the most renowned models of sorption (Freundlich model, Langmuir model, and Temkin model). According to the Langmuir model, on a uniform (homogeneous) surface, monolayer adsorption produces dye adsorption. The Langmuir isothermic model is characterized by condition (3) in which *k_L_* (L/g) is the material’s stable adsorption capacity and adsorption, and *Q*_max_ (mg/g) is the maximum adsorption strength based on the total number of monolayers present. It was a plot against *C_e_* if *C_e_*/*Q_e_* framed a straight line. Using the inclines and blocks, the bounds *Q*_max_ and *k_L_* were established. Within a temperature range of 20 to 60 °C, *Q_e_* max and *k_L_* decreased from 183.0 to 131.1 mg/g, and 0.27 to 0.22 L/g, respectively. Table 1 shows the highest *R*^2^ (0.994) connection values at 20 °C, demonstrating that the Langmuir model may be adjusted to take into account the experiment findings.

(3) $$\frac{C_{e}}{Q_{e}}=\frac{C_{e}}{Q_{max}}+\frac{1}{Q_{max}k_{L}}$$

(4) $$R_{L}=\frac{1}{1+k_{L}C_{e}}$$

This demonstrates that adsorption is favorable when *R_L_* = 0, unfavorable when *R_L_* > 1, linear when *R_L_* = 1, and irreversible when *R_L_* = 0. In the paper, *R_L_* was found to be between 0.062 to 0.11, indicating a successful adsorption process. The following are the ways in which adsorption on a heterogeneous surface affects the linearized Freundlich isotherm adsorption articulation:

(5) $$\ln\;Q_{e}=\ln\;K_{F}+\frac{1}{n}\ln\;C_{e}$$

We use the Freundlich coefficients *K_F_* (L/g) and *n*, which independently display the rate and adsorption power. Plotting ln *Q_e_* vs. ln *C_e_* and determining *n* and *K_F_* from the slope and intercepts resulted in a straight line. The expected values for *n* and *K_F_* were 8.80 to 6.69 and 112.0 to 70.1 L/g, respectively. The third kind is represented by the Temkin isotherm in condition (6) [57].

(6) $$Q_{e}=B\;\ln{\;K}_{t}+B\;\ln{\;C}_{e}$$

The binding constant at equilibrium, *K_t_*, corresponds to the highest possible binding energy. The adsorption pressure and the constant B are connected. When *Q_e_* was plotted against ln *C_e_*, a linear plot was created with a maximum *R*^2^ of 0.754. The Langmuir isotherm correlates better than the other isotherms, according to Table 1’s analysis of the PPy-PEI results for the MB.

### 3.9. Adsorption Kinetics

Using pseudo-first order (PFO) and pseudo-second order (PSO) kinetics, the rate and mechanism of methylene blue dye adsorption were investigated. Comprehensive kinetic of methylene blue adsorption tests at pH 6.2 and almost room temperature of 20 °C were conducted. The (PFO) rate model is expressed by Equation (7).

(7) $$\log\;(Q_{e}-Q_{t})=\log Q_{e}-\frac{k_{1}}{2.303}t$$

The maximal adsorption limit is (mg/g), the steady-state Lagergren scale adsorption is (min^−1^), and the adsorption limit at time in minutes is (mg/g). The values and that were calculated using the graph and the log plot incline (-) are displayed in Table 2. This adsorption procedure does not satisfy the PFO demand mode since the *R*^2^ esteem is poor and the conscious worth is significantly lower than the exploratory worth [58]. Equation (8) describes how to represent the PSO rate model.

(8) $$\frac{t}{Q_{t}}=\frac{1}{k_{2}Q_{e}^{2}}+\frac{t}{Q_{e}}$$

### 3.10. Reusability and Regeneration of the Methylene Blue Adsorbent

**Figure 14:**
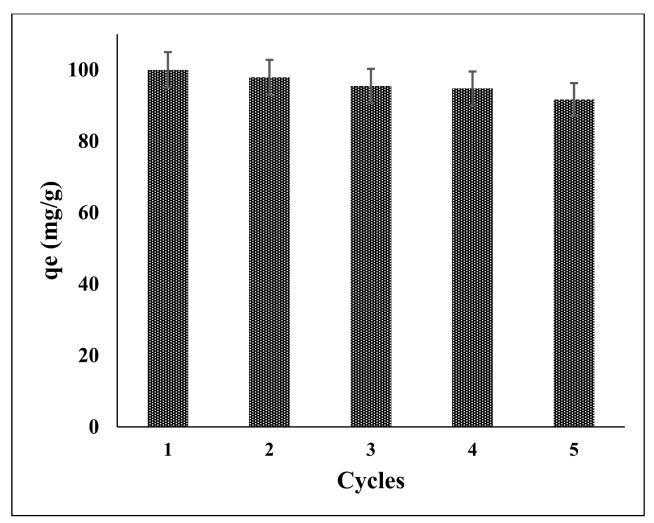
Adsorption capacity of PPy-PEI nano-adsorbent in 5 desorption–adsorption cycles.

The adsorbent’s reusability is critical for its practical application. The recycling experiments were carried out with 0.01 mol/L NaOH solutions for two hours at 180 rpm shaking, producing a desorption efficiency of more than 91 percent after the fifth cycle. The results of five desorption—adsorption tests are shown in Figure 14. The adsorbent’s methylene blue adsorption capability was preserved, indicating that the PPy-PEI adsorbent had high regeneration and reusability.

## 4. Comparative Profile with Previous Literature

In dye adsorption investigations, PPy or its mixture with other substances is employed. Adsorption experiments of Acid orange 7 (AO7) using Polypyrrole/nano silica composite (PPN/SiO_2_) were conducted, with adsorption effectiveness above 99.4%. The PPN/SiO_2_ characteristics were determined using XRD, FTIR, pHzpc, TGA, SEM, and TEM studies. The kinetic data were fit using a PSO kinetic model; the rate constant K increased as temperature increased, indicating that the adsorption was endothermic. The AO7 adsorption process was intricate and regulated by both surface adsorption and particle diffusion, according to the adsorption mechanism investigations. Additionally, the results suggest that for low fixations, adsorption occurs by film dispersion, but at higher fixations, molecule dispersion is the rate-determining step. The Langmuir isotherm was followed by the harmony data, according to the isotherm parameters, with a MAC (maximum adsorption capacity) of 181.4 mg/g [59]. Under optimum conditions (pH 9, starting RR or RB focus, 75 mg/L; PPy/Ze part, 1.8 g/L; temperature, 50 °C), the PPy/Ze nanocomposite absorbed 86.2 percent of RB and 88.3 percent of RR from the designed arrangement. Both RB and RR elimination from synthetic solution were better matched by the Freundlich isotherm model with PSO kinetics [60]. Adsorbents made of PAN/PPy mat were employed to remove cationic yellow dye from a watery solution. The adsorption limit increased when the initial dye focus, adsorption capacity, and temperature increased, reaching a MAC 59.63 mg/g, according to the adsorption findings. Adsorption energy analysis has shown that the PSO model may also be employed to represent the adsorption interaction [61]. During the oxidative polymerization of pyrrole, a conducting polymer called polypyrrole was applied in situ to a cotton substrate (PPy). The resulting materials were used as quickly divisible and reusable adsorbents for the expulsion of methylene blue (MB) in an alkaline medium as a model of cationic dyes with an MAC of 3.30 mg/g. A few factors, including contact time, adsorbent dose, temperature, starting dye concentration, and pH, were investigated for their effects on the adsorption interaction. The adsorption cycle was modeled using the Freundlich isotherm model and the PSO active model. According to the study of thermodynamics, MB adsorption by PPy is a logical, unrestricted, and exothermic cycle. The substrate recovery focus revealed that, taking into account the recovery of methylene blue for reusing, PPy applied to cotton materials could be reused for dye adsorption several times with excellent results [43]. Using pyrrole polymerization with potassium persulfate as an oxidant, a composite made of coffee bean waste (CGW) coated in semi-conducting polypyrrole (PPy) was produced. A SEM analysis of coffee fibers revealed a homogeneous coating of PPy spherical nanoparticles with sizes ranging from 200 to 300 nm. Fluid adsorption studies using the Rhodamine B dye (RhB) were carried out on the composite in its as-arranged state. On adsorption conduct, the effects of pH and initial dye concentration (*C*_0_) were examined. The results demonstrated that at solubility pH, this material was an excellent RhB dye adsorbent. The Redlich–Peterson model and the standard Langmuir adsorption model correctly predicted the RhB adsorption isotherm at a controlled pH of 9.0, with an anticipated MAC of (qmax) of 50.59 mg of dye/g of composite [62]. Table 3 described the utilization of PPy and its combination for dye adsorption under various testing conditions.

## 5. Conclusions and Future Recommendations 

Nano-adsorbents of polypyrrole and polyethyleneimine (PPy-PEI) were effectively produced in this study. In the presence of PEI, pyrrole is chemically oxidized. On the produced nano-adsorbent, cationic methylene blue adsorption from aqueous solution was examined. This work demonstrates that conductive polymers polypyrrole-polyethyleneimine can adsorb the cationic MB dye. At a low temperature (20 °C) and pH of 6.2, the adsorption cycle ought to arrive at equilibrium (with 100% removal) rapidly. The results of the research revealed that the group adsorption parameters, such as adsorption dose, initial pH, and contact duration at room temperature, significantly impacted the viability of methylene blue adsorption. The MAC of the methylene blue dye nano-adsorbents was achieved at a pH of 6.2. By using FTIR and EDX analysis, it has been determined that PPy-PEI contains nitrogen particles, which function as the active site for methylene blue adsorption. Isothermic studies at various temperatures revealed that the Langmuir model could broadly match the exploratory results, in contrast to Freundlich and Temkin’s isotherms. The most extreme limit monolayer, qmax, for methylene blue was found to be 183.3 mg g^−1^ using the Langmuir model. The pseudo-second request was more effective than the pseudo-first request in addressing the adsorption data of the energy at various concentrations. The nano-adsorbent polypyrrole-polyethyleneimine is an excellent choice for use in the removal of the methylene blue dye from an aqueous solution.

Furthermore, the synthesized nano-adsorbent can be used as an efficient and reusable adsorbent for the adsorption of synthetic cationic dyes from textile effluents. Future research should investigate its potential use in treating very acidic industrial wastewater containing organic dyes, for the dual benefit of enhanced adsorption and environmental friendliness. In addition, more research on various modifying agents and synthesis procedures should be conducted with the objective of improving its aqueous phase stability and adsorption capabilities. Additionally, column studies, as well as batch studies, should be utilized to explore the potential of the adsorbent for large-scale applications, and to further implement the removal of industrial dyes from wastewater. Adsorbent adsorption and regeneration research should be prioritized to cut costs and reduce adsorbent waste. As a result, additional studies are needed to create alternative desorption and regeneration processes that would limit the breakdown of conducting polymer adsorbents’ structure and extend their lifespan. Since robust adsorbents are appealing for practical applications, methods to improve the fundamental reliability of leading polymer adsorbents and their composites should be investigated. Some of the studies that were examined provided no information, while others described sophisticated, energy-intensive preparatory processes that could not be accurately duplicated on a commercial scale. Using MB and various hues of overall contaminated waste materials in many research sectors is crucial to reducing the risk of optional tainting caused by the adsorption cycle.

## Figures and Tables

**Table 1:** Demonstrates the parameters of the isotherm model for the ***MB*** adsorption at various temperatures.

Isotherm Model	Parameter	Temperature		
		20 °C	40 °C	60 °C
Langmuir	*qm* (mg/g)	183.3	146.5	131.4
	*k_L_* (L/g)	0.27	0.24	0.22
	*R_L_*	0.062	0.11	0.11
	*R* ^2^	0.994	0.886	0.929
Freundlich	*K_F_* (L/g)	112.0	77.0	70.1
	*N*	8.80	6.68	6.69
	*R* ^2^	0.897	0.845	0.800
Temkin	*A*	0.063	0.192	0.228
	*B*	2.830	2.153	2.473
	*R* ^2^	0.754	0.671	0.725

**Table 2:** Kinetic model parameters for methylene blue adsorption with the increase in initial concentration.

*C*_0_ (mg/L)	*Q_e_* Exp (mg/g)	First-Order Kinetic Model			Second-Order Kinetic Model		
		*Q_e_* Cal (mg/g)	*k*_1_	*R* ^2^	*Q_e_* Cal (mg/g)	*k*_2_	*R* ^2^
10	9.6	32.30	0.069	0.984	64.80	0.0421	0.997
20	19.5	95.02	0.044	0.903	100.30	0.0475	1.00
30	29.4	50.64	0.056	0.987	145.94	0.0972	0.999
40	38.9	17.69	0.060	0.865	172.88	0.3400	0.995

**Table 3:** Comparative profile of polypyrrole based adsorbent for the adsorption of dyes.

Type of Adsorbents	Type of Dyes	Experimental Conditions	Adsorption Capacity	References
Polypyrrole/zeolite (PPy/Ze)	Reactive blue (RB) and reactive red (RR)	(pH 9, initial RR or RB concentration, 75 mg/L; PPy/Ze dose, 1.8 g/L; and temperature, 50 °C).	*Q*max (mg/g) 122.32(RB) 116.53(RR)	[60]
Sodium alginate/polypyrrole	Methylene blue	temperature (25 °C), contact time, initial pH (7), adsorbent dosage (1–5 g/L), dye concentrations (10–50 mg/L).	217.4/666.7 mg/g	[63]
Polypyrrole/sugarcane bagasse, (PPy/SB)	Acid black-234 (AB-234)	pH 3; contact time about 60 min; dose 0.05 g; initial conc. 10 mg/L	100 mg/g	[64]
Polyacrylonitrile/polypyrrole (PAN/PPy)	Yellow dye.	80 °C, 3 h, 30 mg/L	59.63 mg/g	[61]
Polypyrrole/nanosilica composite	Acid orange 7 (AO7)	1 g/L, contact time 90 min, Initial AO7 concentration 10 mg/L, shaking speed = 200 rpm and temperature 328 K and pH = 3)	181.4 mg/g.	[59]
Polypyrrole-coated cotton textile	Methylene blue dye	3.9 mg/L, 45 °C, pH 7400 min,	3.30 mg/g	[43]
Polypyrrole/coffee grounds waste	Rhodamine B dye	200 mg/L, pH 9, 120 min, 25 °C and 125 mg dose	50.59 mg of dye/g	[62]
Polypyrrole nanostructures	Reactive black 5	Dosage 0.2 gm, pH 9 and adsorption time 60 min, Temp 650 °C	100 mg/g	[65]
Polypyrrole	Congo red	Volume of the solution: 150 mL; Initial concentration: 20 mg/L; pH: pH6.55;. Adsorbent dosage: 1.73 g/L. Temperature: 25 °C.	11.53 mg/g	[66]
PPy-PEI nano-adsorbents	Methylene Blue	pH of 6.2; contact time 40 min at room temperature; dose 0.4 g	183 mg/g	Present study

## Data Availability

Not applicable.
